# Integrated analysis of exosome-related genes and their role in psoriasis pathogenesis

**DOI:** 10.3389/fimmu.2025.1492012

**Published:** 2025-06-10

**Authors:** Zhen Wang, Fang Luo

**Affiliations:** ^1^ Department of Dermatology, TaiHe Hospital, The Affiliated Hospital of HuBei University of Medicine, Shiyan, Hubei, China; ^2^ Quality Control Office, TaiHe Hospital, The Affiliated Hospital of HuBei University of Medicine, Shiyan, Hubei, China

**Keywords:** psoriasis, immune response, ERDEGs, gene regulatory networks, PCA

## Abstract

**Objective:**

This study aimed to analyze gene expression data from psoriasis and control samples, focusing on identifying exosome and cell senescence genes, integrating datasets, and validating batch effect removal using principal component analysis (PCA).

**Methods:**

We analyzed gene expression profiles from Gene Expression Omnibus (GEO) to identify significant differences between healthy and diseased tissues. It evaluated immune cell proportion variations and used weighted gene co-expression network analysis (WGCNA) to find key modules. Protein-protein interaction (PPI) networks were constructed to explore gene interactions, followed by enrichment analysis for biological functions and pathways. To validate findings, feature genes were confirmed using additional GEO datasets and real-time fluorescence quantitative PCR (RT-qPCR).

**Results:**

This study integrated GSE30999 and GSE13355 datasets, identifying 274 exosome-related and cell senescence genes. After standardizing and normalizing the data, PCA confirmed effective batch effect removal. Differentially expressed genes (DEGs) were analyzed for immune-related functions, and PPI networks were constructed. The results, visualized with heatmaps, revealed significant differences in the expression of exosome-related DEGs between psoriasis and control samples. These findings provide insights into potential novel targets for psoriasis therapy.

**Conclusions:**

Sixteen exosome-related differentially expressed genes (ERDEGs), including *CD274* and *SERPINB3*, are likely to play a significant role in the development of psoriasis.

## 1 Introduction

Psoriasis is a common chronic and recurrent skin and joint disease mediated by T cells. It is characterized by several features, including hyperproliferation, incomplete keratinization, epidermal thickening, a reduced or absent granular layer, downward extension of the epidermis, inflammatory infiltration, Munro microabscess formation, and dermal microvessel proliferation (1, 2). Excessive keratinocyte proliferation and alterations in the immune microenvironment are interrelated factors that contribute to the development of psoriasis (3). Psoriasis has multifactorial etiology and strong genetic susceptibility, and it can have significant negative impacts on the physical, emotional, and psychological well-being of affected individuals. Psoriasis is found worldwide, although the incidence rates vary among different ethnic groups. The prevalence of psoriasis ranges from 0.14% (95% uncertainty interval: 0.05% to 0.40%) in East Asia to 1.99% (95% uncertainty interval: 0.64% to 6.60%) in Australasia. The prevalence of psoriasis was also high in western Europe (1.92%, 1.07% to 3.46%), central Europe (1.83%, 0.62% to 5.32%), North America (1.50%, 0.63% to 3.60%), and high income southern Latin America (1.10%, 0.36% to 2.96%) (4). The current treatment modalities for psoriasis include topical medications, phototherapy, systemic medications, and physical therapy. Nevertheless, these treatment options come with specific limitations that need to be considered. There can be individual variations in the response and effectiveness of treatments among different patients, and concerns regarding side effects and safety need to be addressed (5). Additionally, the treatments only provide temporary relief, and the efficacy may diminish after discontinuation or in case of relapse. High cost and limited accessibility pose challenges for some patients, and a lack of personalized treatment strategies is evident (6). Therefore, further research and innovation are crucial for developing more effective, safe, and personalized treatment strategies. Additionally, understanding the causes and immune mechanisms of psoriasis can lead to new treatment options and targeted therapies. These advancements aim to improve treatment outcomes, minimize side effects, and enhance patients’ quality of life.

Exosomes are small membrane-bound vesicles that contain various biomolecules, including lipids, proteins, and nucleic acids. Exosomes are derived from cells through exocytosis, are taken up by target cells, and can transmit biological signals between local or distant cells. Exosome secretion is a constant process that occurs in both physiological and pathological contexts. It determines the surface molecules and contents of the exosomes (7, 8). Therefore, exosomes serve as biomarkers, vaccine carriers, and drug delivery vehicles, and can be modified for therapeutic interventions, opening new avenues for effective clinical diagnostics and treatment strategies (9). Gene Ontology (GO) analysis indicated that differentially expressed proteins (DEPs) were primarily linked to keratin filaments, intermediate filaments, extracellular exosomes, the extracellular matrix, the innate immune response, cornification, and keratinocyte differentiation. Kyoto Encyclopedia of Genes and Genomes (KEGG) pathway analysis showed that the estrogen signaling pathway, cholesterol metabolism, fat digestion and absorption, peroxisome proliferator-activated receptors (PPARs), and interleukin-17 (IL-17) signaling pathway might be important pathways in the treatment of psoriasis.

## 2 Materials and methods

### 2.1 Data download

By using the R package (Version 4.3.0) GEO query (10), we downloaded the Psoriasis GSE30999 (11) and GSE13355 (12) datasets from the GEO database (https://www.ncbi.nlm.nih.gov/geo/). The samples in the GSE30999 and GSE13355 datasets were all derived from Homo sapiens, with skin as the tissue source. The chip platform used in these datasets was GPL570, as detailed in **Table 1**. Among them, GSE30999 contained 85 Psoriasis samples and 85 Control samples, while GSE13355 contained 58 Psoriasis samples and 122 Control samples. All Psoriasis samples and Control samples were included in this study. We collected cell aging-related genes, including Cellular Senescence-Related Genes (CSRGs), from the GeneCards database (13) (https://www.genecards.org/) and published literature. The GeneCards database provides comprehensive information on human genes. After using the term “Exosome” as a search keyword and keeping only exosome-associated genes (ERGs) with “Protein Coding” and “Relevance Score > 3,” a total of 158 ERGs were obtained. Likewise, we used “Exosome” as keywords on the PubMed website (https://pubmed.ncbi.nlm.nih.gov/) to identify cell aging-related genes in published literature (14), resulting in a total of 121 ERGs. A total of 274 ERGs were obtained after merging and removing duplicates, and detailed information is shown in **Supplementary Table S1**. The R package sva (15) was used to remove batch effects from GSE30999 and GSE13355 to obtain the Combined GEO Datasets. The Combined Datasets included 143 Psoriasis samples and 207 Control samples. Finally, the R package limma (16) was used to standardize the Combined GEO Datasets, annotate probes, and normalize them. The expression matrices before and after batch effect removal were subjected to Principal Component Analysis (PCA) (17) to verify the effectiveness of this process. PCA is a data dimensionality reduction method that extracts the feature vectors (components) of data from high-dimensional data, transforming it into low-dimensional data and displaying these features in 2D or 3D graphs.

**Table 1:** GEO microarray chip information.

Characteristics	GSE30999	GSE13355
Platform	GPL570	GPL570
Type	Array	Array
Species	Homo sapiens	Homo sapiens
Tissue	Skin	Skin
Samples in Disease group	85	58
Samples in Control group	85	122
Reference	PMID: 22763790	PMID: 21483750

GEO, Gene Expression Omnibus.

### 2.2 Differentially expressed genes in psoriasis-related exosomes

The Combined GEO Datasets categorized samples into the Psoriasis group and the Control group. The R package limma was used to perform differential analysis of genes between the Psoriasis group and the Control group. For the threshold of DEGs, we set |logFC| > 1 and adjusted *p* < 0.05. Genes with logFC > 1 and adjusted *p* < 0.05 were classified as up-regulated genes, while genes with logFC < -1 and adjusted *p* < 0.05 were classified as down-regulated genes. The method used for adjusting *p*-values was the Benjamini-Hochberg procedure. The results of the difference analysis were plotted by volcano plot through the R package ggplot2.

We integrate the GEO dataset to identify ERDEGs related to Psoriasis. A dataset is derived from variance analysis, selecting genes with |logFC| > 1 and adjusted *p* < 0.05. We identify DEGs associated with external secretion (ERGs) and determine their intersection to establish ERDEGs. This is visualized using Venn diagrams and heat maps generated by the R packages pheatmap and RCircos (18).

### 2.3 Gene ontology and pathway analysis (KEGG) were performed

GO analysis (19)is a widely used method for large-scale studies of functional enrichment, focusing on Biological Processes (BP), Cellular Components (CC), and Molecular Functions (MF). Similarly, the KEGG (20) is a popular database that provides information on genomes, biological pathways, diseases, and drugs. GO and KEGG pathway enrichment analyses were conducted using the R package clusterProfiler (21) for ERDEGs. The criteria for statistical significance included a *p*-value of less than 0.05 and a false discovery rate (FDR) of less than 0.25.

### 2.4 Gene set enrichment analysis

GSEA (22) evaluates how genes are distributed in a predefined gene set, which is ranked based on their correlation with a phenotype. This analysis helps determine the genes’ contributions to that phenotype. In this study, we first ranked the genes from the Combined GEO Datasets by their logFC values. Then, we used the R package clusterProfiler to perform GSEA on all genes in the integrated GEO Datasets. The parameters used in the GSEA were as follows: the seed was set to 2023, with a minimum of 10 genes and a maximum of 500 genes per gene set. We accessed the c2 gene sets from the Molecular Signatures Database (MSigDB), specifically using the Cp. All. V2022.1. Hs. Symbols. The GMT file containing all Canonical Pathways (3050) was utilized for the GSEA. The GSEA screening criteria were an adjusted *p*-value of less than 0.05 and a false discovery rate (FDR) value (*q*-value) of less than 0.25, with *p*-value correction applied using the Benjamini-Hochberg method.

### 2.5 Protein-protein interaction network and hub gene screening

The PPI network is a crucial tool for understanding how proteins interact with one another and their roles in biological processes. In this study, we utilized the STRING database (23)(https://string-db.org/)to analyze the interactions of ERDEGs. This database offers known protein-protein interactions and predicts potential ones using various evidence sources, such as experimental data and computational predictions.

We constructed a PPI network for the 16 ERDEGs identified in our study. The network was built using a minimum interaction score greater than 0.15, which represents a low confidence level but allows for a broader exploration of potential interactions (24). The resulting network helps to visualize the complex web of interactions among these genes and may reveal molecular complexes or pathways that are relevant to psoriasis. To identify the most significant genes within the PPI network (referred to as Hub Genes), we employed five algorithms from the CytoHubba plug-in of Cytoscape software (25):Maximal Clique Centrality (MCC), Degree, Maximum Neighborhood Component (MNC), Edge Percolated Component (EPC), and Closeness. These algorithms help to score and rank genes based on their connectivity and importance within the network.

The top 10 genes from each algorithm were selected and compared. A Venn diagram was created to identify the intersection genes that were consistently ranked highly across all algorithms. These intersection genes were identified as exosome-related Hub Genes, likely playing central roles in the pathogenesis of psoriasis.

### 2.6 Construction of regulatory network

Transcription factors (TFs) control gene expression through interactions with Hub Genes at the post-transcriptional stage. We utilized the ChIPBase database (26) (https://rnasysu.com/chipbase3/index.php) to retrieve transcription factors, applying a filter condition where the number of samples found (both upstream and downstream) is greater than 9. The regulatory effects of transcription factors on Hub Genes were analyzed, and the mRNA-TF regulatory network was visualized using Cytoscape software. Additionally, miRNAs play a crucial regulatory role in biological development and evolution. They can regulate a variety of target genes, and the same target gene can also be regulated by multiple miRNAs. To analyze the Hub Genes and their relationship with miRNAs, we accessed the StarBase v3.0 database (27)(https://rnasysu.com/encori/) to identify miRNAs associated with the Hub Genes. We selected mRNA-miRNA interaction relationships recorded from at least two sources and visualized the mRNA-miRNA regulatory network using Cytoscape software.

### 2.7 Hub gene expression differences and ROC curve analysis

To examine the differences in Hub Gene expression between the Psoriasis and Control groups, we created a comparison map based on their expression levels. To further analyze the differences in Hub Gene expression between the Psoriasis and Control groups in the GEO Datasets, we created a comparison map. Finally, we used the R package pROC to plot the ROC curve for the Hub Genes and calculate the Area Under the Curve (AUC). This analysis aimed to evaluate how Hub Gene expression affects the occurrence of Psoriasis. The AUC of the ROC curve typically ranges from 0.5 to 1, with values closer to 1 indicating better diagnostic performance.

### 2.8 Immune infiltration analysis

Single-Sample Gene-Set Enrichment Analysis (ssGSEA) (28) quantifies the relative abundance of each immune cell infiltrate. First, the types of infiltrating immune cells were labeled, including Activated Dendritic Cells, Gamma Delta T Cells, Natural Killer Cells, and Activated CD8^+^ T Cells. Next, the R package ggplot2 was employed to create comparison maps illustrating the expression differences of immune cells between the Control and Psoriasis groups in the Combined GEO Datasets. Following this, the R package ggplot2 was utilized to generate comparative plots that illustrate the variances in immune cell expression between the Control and Psoriasis groups in the Combined GEO Datasets. The correlation between immune cells was calculated using the Spearman algorithm. The R package pheatmap was then employed to create a heatmap displaying the correlation analysis results of the immune cells. The correlation between Hub Genes and immune cells was also calculated using the Spearman algorithm, and a correlation bubble plot was drawn using the R package ggplot2 to show the correlation analysis results of Hub Genes and immune cells.

### 2.9 Statistical analysis

All data processing and analysis in this article were based on R software (Version 4.3.0). To compare continuous variables between two groups, we used the independent Student’s T-Test for normally distributed variables, unless stated otherwise. We applied the Mann-Whitney U Test (Wilcoxon Rank Sum Test) to analyze differences in non-normally distributed variables. We used the Kruskal-Wallis test to compare three or more groups. We used Spearman correlation analysis to assess the correlation coefficient between different molecules. All p-values were two-sided unless specified otherwise. A *p*-value of less than 0.05 was considered statistically significant.

## 3 Results

### 3.1 Technology roadmap

**Figure 1:**
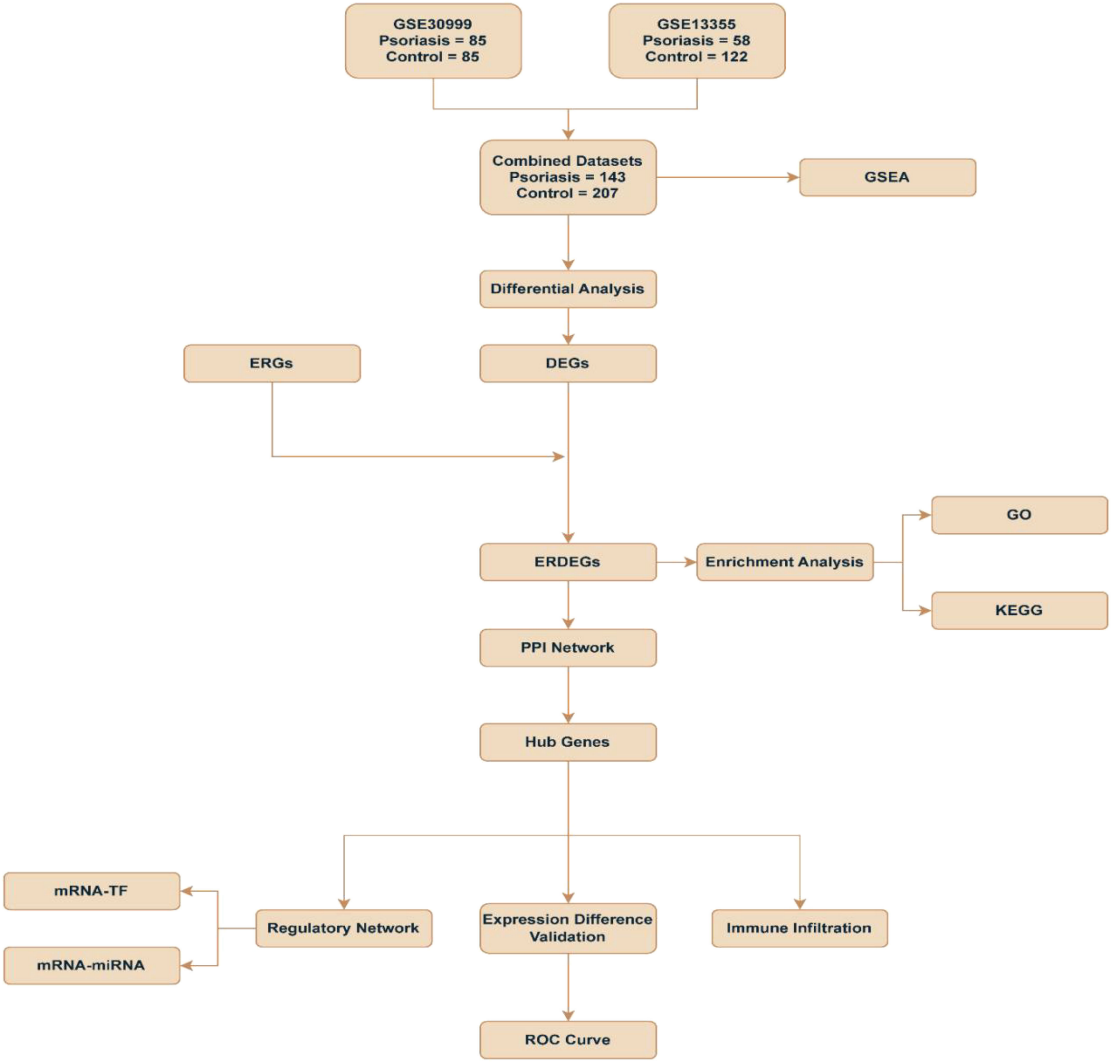
Flow chart for the comprehensive analysis of ERDEGs. DEGs, Differentially Expressed Genes; ERGs, Exosome-Related Genes; ERDEGs, Exosome-Related Differentially Expressed Genes; ROC, Receiver Operating Characteristic; GSEA, Gene Set Enrichment Analysis; GO, Gene Ontology; KEGG, Kyoto Encyclopedia of Genes and Genomes; PPI, Protein-Protein Interaction; TF, Transcription Factors.

This study combines two datasets: GSE13355, which includes 58 psoriasis patients and 122 controls, and GSE30999, which consists of 85 psoriasis patients and 85 controls. It aims to systematically identify DEGs in psoriasis and explore their biological functions using GSEA. Additionally, a PPI network was constructed to identify key hub genes. Regulatory networks involving mRNA-TF and mRNA-miRNA were also analyzed to reveal potential transcriptional regulatory mechanisms.We conducted functional enrichment analysis (GO and KEGG), ROC curve assessment, and immune infiltration analysis to evaluate the diagnostic value of these genes in psoriasis. This comprehensive evaluation sheds light on their roles in the immune microenvironment and offers a new perspective on psoriasis pathogenesis and potential biomarkers (**Figure 1**).

### 3.2 Merging of psoriasis datasets

**Figure 2:**
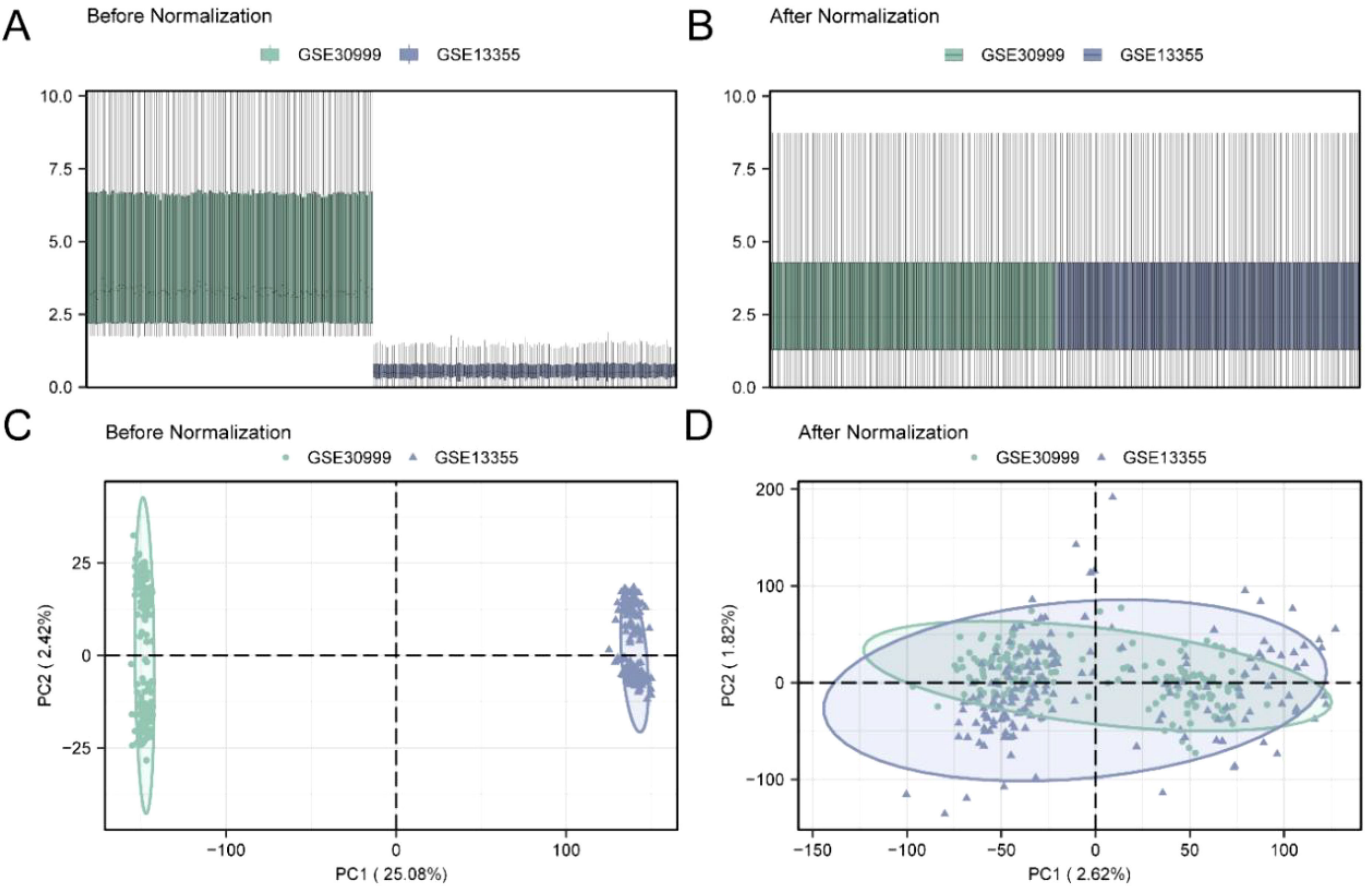
Batch effects removal of GSE30999 and GSE13355. **(A)** Box plot of Combined GEO Datasets distribution before batch removal. **(B)** Post-batch integrated GEO Datasets (Combined Datasets) distribution boxplots. **(C)** 2D PCA plot of the datasets before debatching. **(D)** 2D PCA plots of Combined GEO Datasets after debatching. PCA, Principal Component Analysis. The psoriasis dataset GSE30999 is shown in green and the psoriasis dataset GSE13355 is shown in blue.

The R package sva was used to remove batch effects from the GSE30999 and GSE13355 datasets, resulting in a combined GEO dataset. First, we used a distribution boxplot (**Figures 2A, B**) to compare the expression values of the datasets before and after batch effect removal. Next, we employed a PCA plot (**Figures 2C, D**) to compare the distribution of low-dimensional features before and after the removal of batch effects. The results from the distribution boxplot and PCA plot indicated that the batch effects in the psoriasis dataset were effectively eliminated following the removal process.

### 3.3 Psoriasis-related exosome-related differentially expressed genes

**Figure 3:**
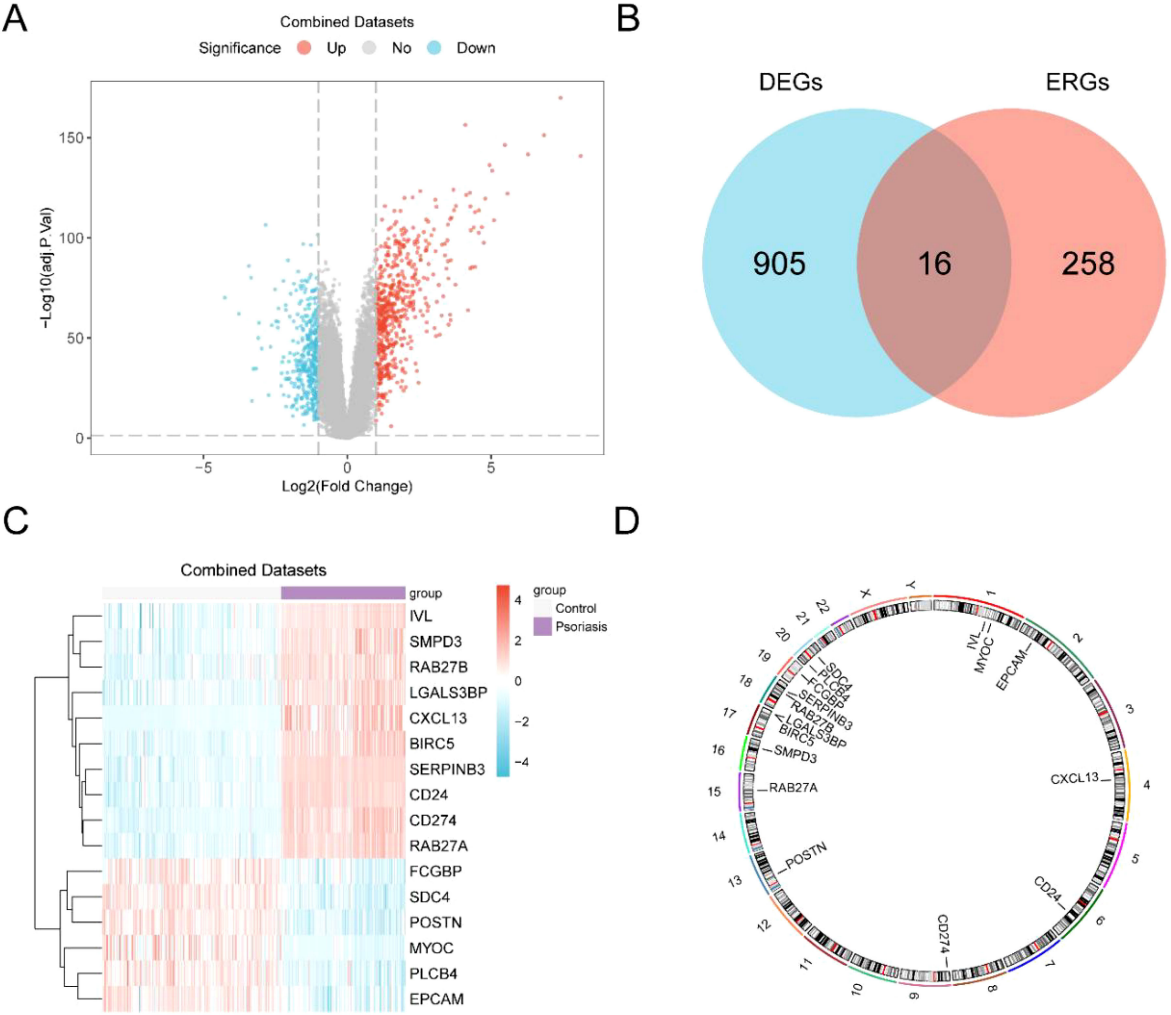
Differential gene expression analysis. **(A)** Volcano plot of differentially expressed genes analysis between Psoriasis group and Control group in the Combined GEO Datasets. **(B)** Differentially expressed genes (DEGs) and exosome-associated genes (ERGs) Venn diagram in the integrated GEO Datasets (Combined Datasets). **(C)** Heat map of exosomes-associated differentially expressed genes (ERDEGs) in the integrated GEO Datasets (Combined Datasets). **(D)** Chromosomal mapping of exosomes-associated differentially expressed genes (ERDEGs). DEGs, Differentially Expressed Genes; ERGs, Exosome-Related Genes; ERDEGs, Exosome-Related Differentially Expressed Genes. Purple is the Psoriasis group, and gray is the Control group. In the heat map, red represents high expression, blue represents low expression, and the depth of color represents the degree of expression.

The Combined GEO Datasets were split into two groups: psoriasis and control. We used the R package limma to analyze gene expression differences between the psoriasis and control groups in the Combined GEO Datasets. The results are as follows: In the Integrated GEO Dataset (Combined Datasets), a total of 921 differentially expressed genes (DEGs) met the thresholds of |logFC| > 1 and adjusted p < 0.05. According to the variance analysis results shown in the volcano plot (**Figure 3A**), there are 555 upregulated genes (logFC > 1 and adj. P < 0.05) and 366 downregulated genes (logFC < 1 and adj. P < 0.05). We identified ERDEGs by intersecting DEGs that met the criteria of |logFC| > 1 and adjusted p < 0.05 with known ERGs, as displayed in the Venn diagram (**Figure 3B**).We identified 16 ERDEGs, which are: *CD274, SERPINB3, CD24, RAB27A, BIRC5, CXCL13, SDC4, LGALS3BP, POSTN, SMPD3, RAB27B, IVL, PLCB4, EPCAM, FCGBP*, and *MYOC*. According to the intersection results, the expression differences of ERDEGs between different sample groups in the Combined GEO Datasets were analyzed, and the R package pheatmap was used to create a heatmap to display the analysis results (**Figure 3C**). Finally, the location of the 16 ERDEGs on human chromosomes was analyzed using the R package RCircos to draw a chromosome localization map (**Figure 3D**). Chromosome mapping revealed that most ERDEGs were found on chromosomes 1, 17, 18, and 20. Specifically, *IVL* and *MYOC* are on chromosome 1, *BIRC5* and *LGALS3BP* are on chromosome 17, *RAB27B* and *SERPINB3* are on chromosome 18, and *PLCB4* and *SDC4* are on chromosome 20.

### 3.4 Gene ontology and Kyoto Encyclopedia of Genes and Genomes analysis

**Figure 4:**
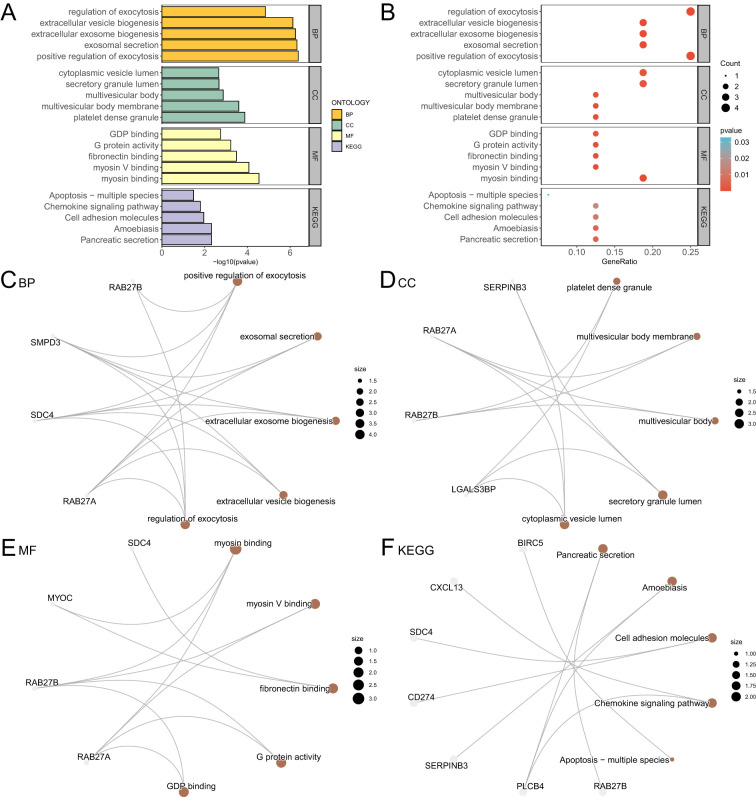
GO and KEGG enrichment analysis for ERDEGs. **(A, B)** Gene ontology (GO) and pathway (KEGG) enrichment analysis results of exosom-related differentially expressed genes (ERDEGs) Bar graph **(A)** and bubble diagram **(B)** show: biological process (BP), cellular component (CC), molecular function (MF) and biological pathway (KEGG). GO terms and KEGG terms are shown on the ordinate. **(C-F)** Gene ontology (GO) and pathway (KEGG) enrichment analysis results of exosomes-associated differentially expressed genes (ERDEGs) network diagram showing BP **(C)**, CC **(D)**, MF **(E)** and KEGG **(F)**. The brown nodes represent items, the gray nodes represent molecules, and the lines represent the relationship between items and molecules. ERDEGs, Exosome-Related Differentially Expressed Genes; GO, Gene Ontology; KEGG, Kyoto Encyclopedia of Genes and Genomes; BP, Biological Process; CC, Cellular Component; MF, Molecular Function. The bubble size in the bubble plot represents the number of genes, and the color of the bubble represents the size of the p value. The more red the color, the smaller the p value, and the more blue the p value. The screening criteria for gene ontology (GO) and pathway (KEGG) enrichment analysis were p value < 0.05 and FDR value (q value) < 0.25.

We performed GO and KEGG analyses to explore the relationships between BP, CC, MF, and pathways related to 16 ERDEGs in psoriasis. We used the 16 ERDEGs for GO and KEGG, with specific results presented in **Table 2**. The results indicated that the 16 differentially expressed early response genes (ERDEGs) in psoriasis were primarily enriched in processes such as positive regulation of exocytosis, exosomal secretion, extracellular exosome biogenesis, and extracellular vesicle biogenesis. The analysis also revealed enrichment in several KEGG biological pathways, including pancreatic secretion, amoebiasis, cell adhesion molecules, chemokine signaling pathways, and apoptosis in various species. The results of the GO and KEGG enrichment analyses are illustrated using bar graphs and bubble plots (**Figures 4A, B**).Additionally, a network diagram illustrating biological processes (BP), cellular components (CC), molecular functions (MF), and KEGG pathways was generated based on the GO and KEGG enrichment analyses (**Figures 4C–F**).The lines illustrate the corresponding molecules and annotations of the respective entries, with larger nodes indicating a greater number of molecules contained within those entries.

**Table 2:** Result of GO and KEGG enrichment analysis for ERDEGs.

ONTOLOGY	ID	Description	Gene Ratio	Bg Ratio	p value	p.adjust	q value
BP	GO:0045921	positive regulation of exocytosis	4/15	80/18614	4.16 e-07	4.16 e-07	7.13 e-05
BP	GO:1990182	exosomal secretion	3/15	20/18614	4.79 e-07	4.79 e-07	7.13 e-05
BP	GO:0097734	extracellular exosome biogenesis	3/15	21/18614	5.58 e-07	5.58 e-07	7.13 e-05
BP	GO:0140112	extracellular vesicle biogenesis	3/15	23/18614	7.43 e-07	7.43 e-07	7.13 e-05
BP	GO:0017157	regulation of exocytosis	4/15	194/18614	1.43 e-05	1.43 e-05	1.10 e-03
CC	GO:0042827	platelet dense granule	2/16	21/19518	1.31 e-04	1.31 e-04	7.18 e-03
CC	GO:0032585	multivesicular body membrane	2/16	29/19518	2.53 e-04	2.53 e-04	7.18 e-03
CC	GO:0005771	multivesicular body	2/16	67/19518	1.35 e-03	1.35 e-03	2.10 e-02
CC	GO:0034774	secretory granule lumen	3/16	322/19518	2.12 e-03	2.12 e-03	2.10 e-02
CC	GO:0060205	cytoplasmic vesicle lumen	3/16	325/19518	2.18 e-03	2.18 e-03	2.10 e-02
MF	GO:0017022	myosin binding	3/16	70/18369	2.86 e-05	2.86 e-05	9.65 e-04
MF	GO:0031489	myosin V binding	2/16	16/18369	8.48 e-05	8.48 e-05	1.43 e-03
MF	GO:0001968	fibronectin binding	2/16	31/18369	3.26 e-04	3.26 e-04	3.66 e-03
MF	GO:0003925	G protein activity	2/16	42/18369	6.00 e-04	6.00 e-04	5.05 e-03
MF	GO:0019003	GDP binding	2/16	73/18369	1.80 e-03	1.80 e-03	1.21 e-02
KEGG	hsa04972	Pancreatic secretion	2/9	102/8659	4.69 e-03	4.69 e-03	1.70 e-01
KEGG	hsa05146	Amoebiasis	2/9	102/8659	4.69 e-03	4.69 e-03	1.70 e-01
KEGG	hsa04514	Cell adhesion molecules	2/9	158/8659	1.09 e-02	1.09 e-02	1.87 e-01
KEGG	hsa04062	Chemokine signaling pathway	2/9	192/8659	1.59 e-02	1.59 e-02	1.87 e-01
KEGG	hsa04215	Apoptosis - multiple species	1/9	32/8659	3.28 e-02	3.28 e-02	1.87 e-01

GO, Gene Ontology; BP, Biological Process; CC, Cellular Component; MF, Molecular Function; KEGG, Kyoto Encyclopedia of Genes and Genomes; ERDEGs, Exosome-Related Differentially Expressed Genes.

### 3.5 Gene set enrichment analysis

**Figure 5:**
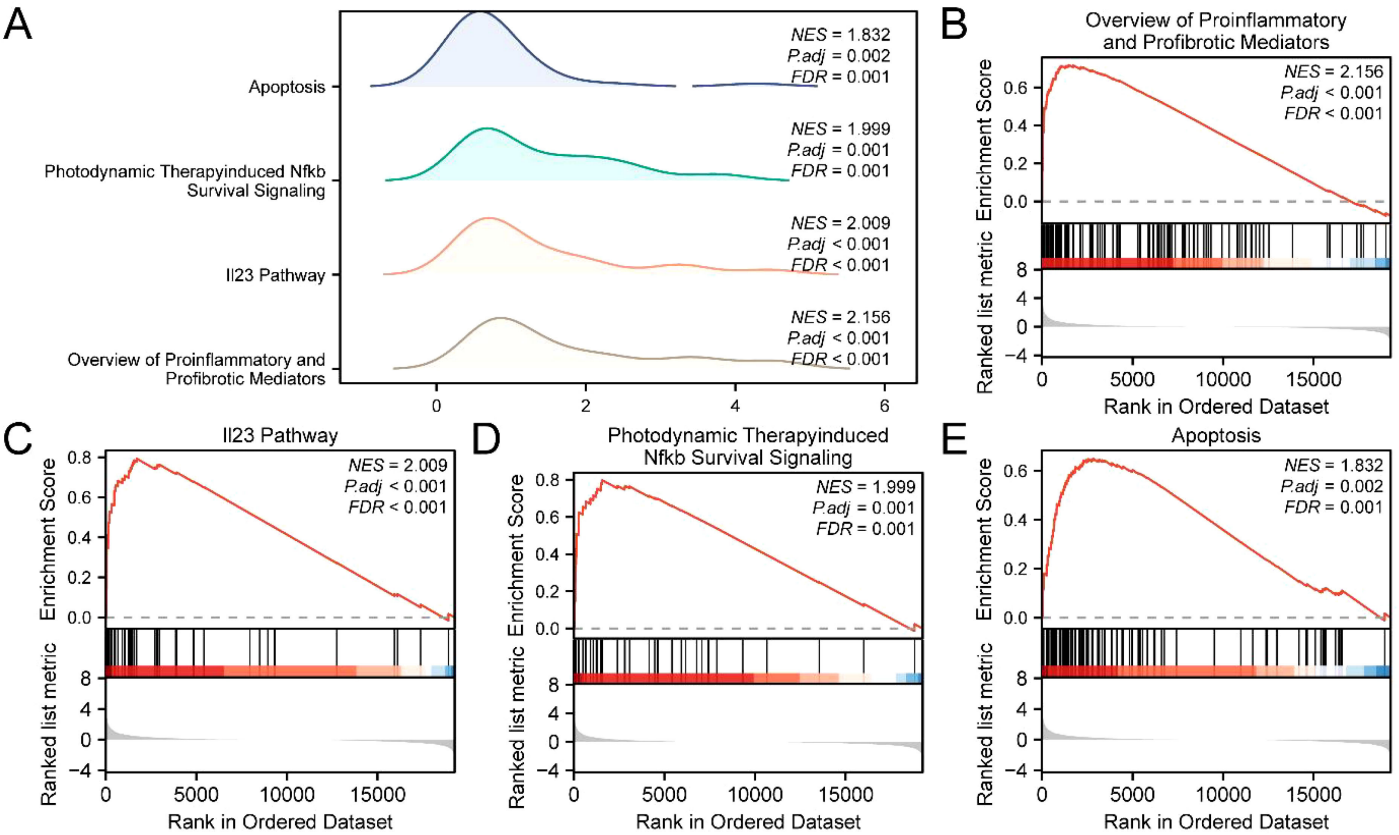
GSEA for combined datasets. **(A)** Gene set enrichment analysis (GSEA) 4 biological functions mountain map presentation of the Combined GEO Datasets. **(B–E)** gene set enrichment analysis (GSEA) showed a significant enrichment in all genes in the Overview of Proinflammatory and Profibrotic Mediators **(B)**, Il23 Pathway **(C)**, Photodynamic Therapyinduced Nfkb Survival Signaling **(D)** and Apoptosis **(E)**. GSEA, Gene Set Enrichment Analysis; ERDEGs, Exosome-Related Differentially Expressed Genes; NES, Normalized Enrichment Score; FDR, False Discovery Rate. The screening criteria of gene set enrichment analysis (GSEA) were adj.p < 0.05 and FDR value (q value) < 0.25, and the p value correction method was Benjamini-Hochberg (BH).

We used GSEA to assess how gene expression levels in the Combined GEO Datasets impact Psoriasis. This analysis explored the relationships between gene expression and associated biological processes, affected cellular components, and molecular functions (**Figure 5A**). The results indicate that the integration of the Combined GEO Datasets reveals significant enrichment in pro-inflammatory and profibrotic mediators (**Figure 5B**), the IL-23 pathway (**Figure 5C**), photodynamic therapy-induced NF-κB survival signaling (**Figure 5D**), apoptosis (**Figure 5E**), and other related biological functions and signaling pathways.

The GSEA analysis results reveal several biological mechanisms related to psoriasis, including the interferon signaling pathway, antimicrobial peptides, SARS-CoV-2 signaling, pro-inflammatory and profibrotic mediators, and cell cycle checkpoints.These findings offer valuable insights into the pathogenesis of psoriasis and suggest potential targets for future research and treatment (**Table 3**).

**Table 3:** Results of GSEA for combined datasets.

ID	Set Size	Enrichment Score	NES	*p* value	*p*.adjust	*q* value
REACTOME_INTERFERON_ALPHA_BETA_SIGNALING	70	0.82085	2.27002	1.00 e-10	2.77 e-08	2.24 e-08
REACTOME_ANTIMICROBIAL_PEPTIDES	65	0.78103	2.16143	1.91 e-08	3.17 e-06	2.57 e-06
WP_SARSCOV2_INNATE_IMMUNITY_EVASION_AND_CELLSPECIFIC_IM MUNE_RESPONSE	64	0.78126	2.15692	4.09 e-08	6.38 e-06	5.17 e-06
WP_OVERVIEW_OF_PROINFLAMMATORY_AND_PROFIBROTIC_MEDIATORS	117	0.71894	2.15550	3.31 e-10	7.49 e-08	6.07 e-08
REACTOME_INTERFERON_SIGNALING	189	0.67549	2.15101	1.00 e-10	2.77 e-08	2.24 e-08
WP_NETWORK_MAP_OF_SARSCOV2_SIGNALING_PATHWAY	210	0.66628	2.14520	1.00 e-10	2.77 e-08	2.24 e-08
REACTOME_INTERLEUKIN_10_SIGNALING	43	0.82527	2.11725	7.04 e-08	9.74 e-06	7.90 e-06
REACTOME_CELL_CYCLE_CHECKPOINTS	270	0.64278	2.11263	1.00 e-10	2.77 e-08	2.24 e-08
REACTOME_RESOLUTION_OF_SISTER_CHROMATID_COHESION	119	0.69019	2.06578	1.04 e-08	1.99 e-06	1.61 e-06
WP_PROSTAGLANDIN_SIGNALING	32	0.83084	2.05134	4.70 e-06	3.08 e-04	2.50 e-04
WP_MIRNAS_INVOLVEMENT_IN_THE_IMMUNE_RESPONSE_IN_SEPSIS	36	0.81196	2.04356	4.63 e-06	3.08 e-04	2.50 e-04
REACTOME_MITOTIC_METAPHASE_AND_ANAPHASE	222	0.63044	2.03761	2.57 e-10	6.39 e-08	5.18 e-08
KEGG_NOD_LIKE_RECEPTOR_SIGNALING_PATHWAY	61	0.74389	2.03232	1.10 e-06	9.45 e-05	7.66 e-05
REACTOME_MITOTIC_SPINDLE_CHECKPOINT	108	0.68401	2.01556	1.27 e-07	1.58 e-05	1.28 e-05
PID_IL23_PATHWAY	37	0.79333	2.00891	8.73 e-06	4.83 e-04	3.92 e-04
REACTOME_SEPARATION_OF_SISTER_CHROMATIDS	179	0.63632	2.00706	5.78 e-09	1.20 e-06	9.73 e-07
REACTOME_DNA_REPLICATION	158	0.64387	2.00623	1.36 e-08	2.42 e-06	1.96 e-06
REACTOME_CHEMOKINE_RECEPTORS_BIND_CHEMOKINES	54	0.74510	2.00195	6.31 e-06	3.83 e-04	3.11 e-04
WP_PHOTODYNAMIC_THERAPYINDUCED_NFKB_SURVIVAL_SIGNALING	35	0.79784	1.99926	3.45 e-05	1.35 e-03	1.09 e-03
WP_APOPTOSIS	82	0.65008	1.83235	4.18 e-05	1.55 e-03	1.26 e-03

GSEA, Gene Set Enrichment Analysis.

### 3.6 Construction of protein-protein interaction network and screening of hub genes

**Figure 6:**
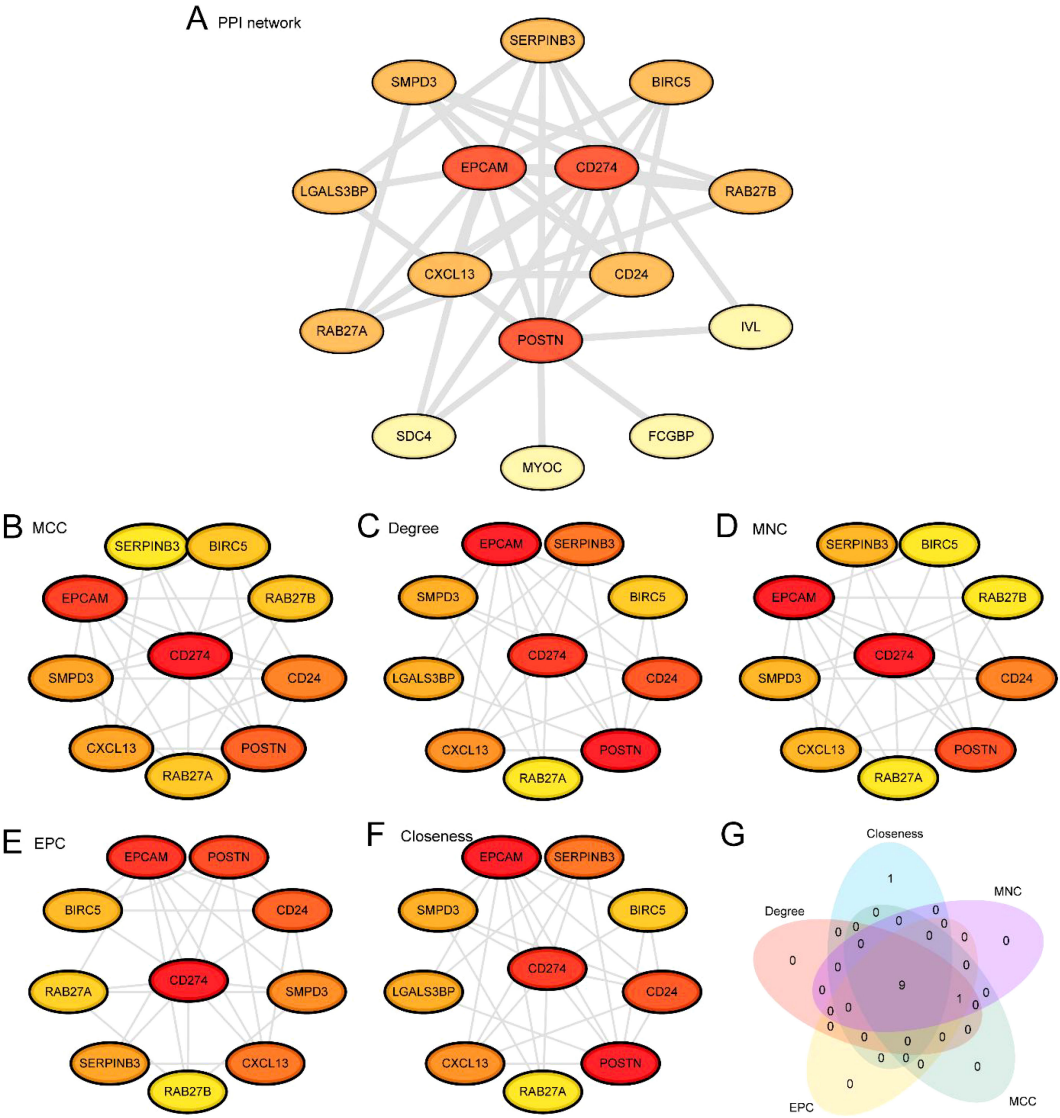
PPI network and hub genes analysis. **(A)** Protein-protein interaction Network (PPI Network) of exosome-related differentially expressed genes (ERDEGs) calculated from STRING database. B-F. Protein-protein interaction Network (PPI Network) of TOP10 exosomes-associated differentially expressed genes (ERDEGs) calculated by 5 algorithms of CytoHubba plug-in, including MCC **(B)**, Degree **(C)**, MNC **(D)**, EPC **(E)** and Closeness **(F)**. **(G)** Exosome-associated differentially expressed genes (ERDEGs) Venn diagram of TOP10 for the 5 algorithms of the CytoHubba plugin. PPI Network, Protein-protein Interaction Network; ERDEGs, Exosome-Related Differentially Expressed Genes.

First, we conducted a protein-protein interaction analysis and constructed a PPI network of 16 ERDEGs using the STRING database (**Figure 6A**). The results of the PPI network indicated that 15 ERDEGs were related: *CD274, SERPINB3, CD24, RAB27A, BIRC5, CXCL13, SDC4, LGALS3BP, POSTN, SMPD3, RAB27B, IVL, EPCAM, FCGBP*, and *MYOC.* Next, we used five algorithms from the CytoHubba plug-in of Cytoscape software to calculate the scores of the 15 ERDEGs. The ERDEGs were then ranked based on these scores. The five algorithms used were: Maximal Clique Centrality (MCC), Degree, Maximum Neighborhood Component (MNC), Edge Percolated Component (EPC), and Closeness. Then, the top 10 ERDEGs from the five algorithms were used to draw protein-protein interaction networks, namely MCC (**Figure 6B**), MNC (**Figure 6C**), Degree (**Figure 6D**), EPC (**Figure 6E**), and Closeness (**Figure 6F**). In this representation, the circle colors range from red to yellow, indicating scores from high to low. Finally, the genes from the five algorithms were interfaced, and a Venn diagram (**Figure 6G**) was drawn for analysis. The intersection genes of the algorithms were exosome-related hub genes, and the 9 hub genes were: *POSTN*, *EPCAM, CD274, CD24, SERPINB3, CXCL13, SMPD3, BIRC5*, and *RAB27A.*

### 3.7 Construction of regulatory networks

**Figure 7:**
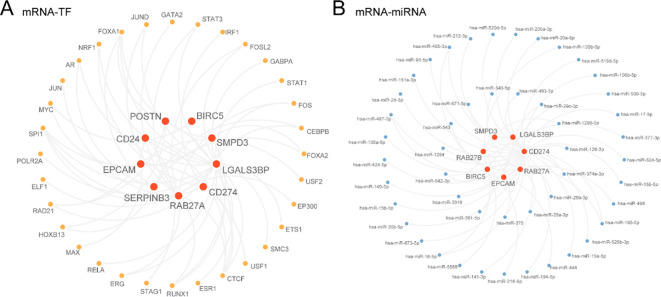
Regulatory network analysis of hub genes**. (A, B)** mRNA-TF Regulatory Network **(A)** and mRNA-miRNA Regulatory Network **(B)** of Hub Genes. TF, Transcription Factors. Red shows Hub Genes (Hub Genes), orange shows transcription factors (TFS), and blue shows mirnas.

First, we obtained TFs associated with Hub Genes from the ChIPBase database, using a screening criterion of more than 9 samples found (upstream + downstream). We constructed and visualized the mRNA-TF Regulatory Network using Cytoscape software (**Figure 7A**). This included 9 Hub Genes and 32 TFs, with specific information provided in **Supplementary Table S2**. Next, we identified miRNAs related to Hub Genes from the StarBase database, using screening criteria that required mRNA-miRNA interaction relationships to be recorded from at least two sources. The mRNA-miRNA Regulatory Network was constructed and visualized by Cytoscape software (**Figure 7B**).This analysis identified 7 Hub Genes and 48 miRNAs, with detailed information available in **Supplementary Table S3**.

### 3.8 Hub gene expression differences and ROC curve analysis

**Figure 8:**
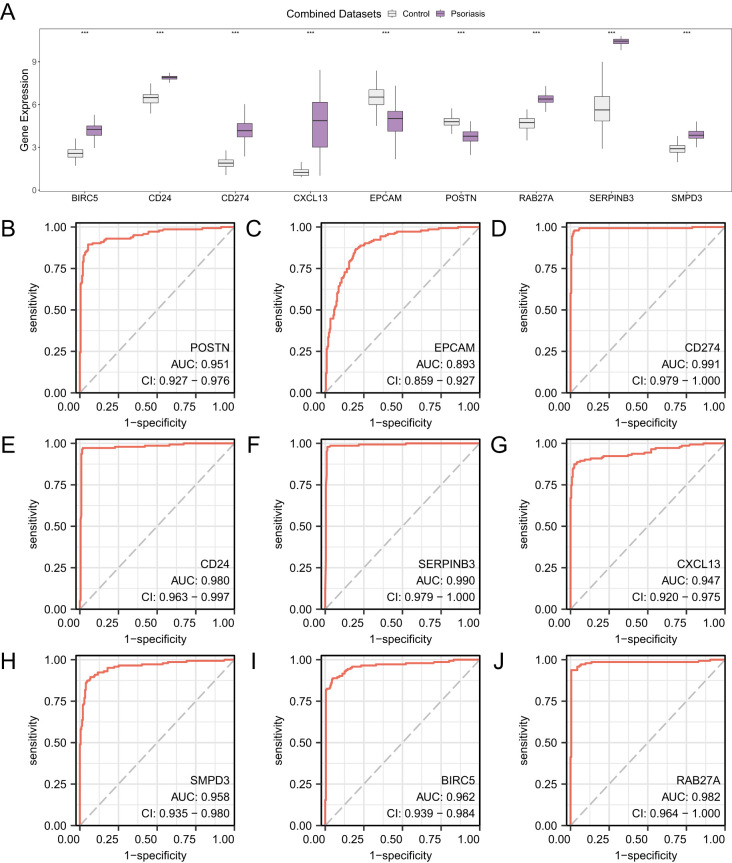
Differential expression validation and ROC curve analysis. **(A)** Group comparison of Hub Genes in the Psoriasis group and the Control group. **(B-J)** ROC curves of Hub Genes in Combined GEO Datasets. *** represents a p value < 0.001 and highly statistically significant. When AUC > 0.5, it indicates that the expression of the molecule is a trend to promote the occurrence of the event, and the closer the AUC is to 1, the better the diagnostic effect. AUC between 0.7 and 0.9 had a certain accuracy, and AUC above 0.9 had a high accuracy. In the group comparison diagram, purple is the Psoriasis group, and gray is the Control group.

**Figure 8A** illustrates the differential expression of nine Hub Genes in the Combined GEO Datasets, comparing the Psoriasis group to the Control group. The results indicate that the expression levels of nine Hub Genes in the Psoriasis and Control groups of the Combined GEO Datasets were highly statistically significant (*p* < 0.001): *POSTN, EPCAM, CD274, CD24, SERPINB3, CXCL13, SMPD3, BIRC5*, and *RAB27A*. Finally, the R package pROC was used to generate ROC curves based on the expression levels of Hub Genes in the Combined GEO Datasets. The ROC curves (**Figures 8B–J**) demonstrated that the expression levels of eight Hub Genes—P*OSTN, CD274, CD24, SERPINB3, CXCL13, SMPD3, BIRC5*, and *RAB27A*—exhibited high accuracy (AUC > 0.9) in classifying the Psoriasis and Control groups. The expression level of EPCAM demonstrated moderate accuracy (0.7 < AUC < 0.9) in distinguishing between the Psoriasis and Control. groups.

### 3.9 Analysis of immune infiltration

**Figure 9:**
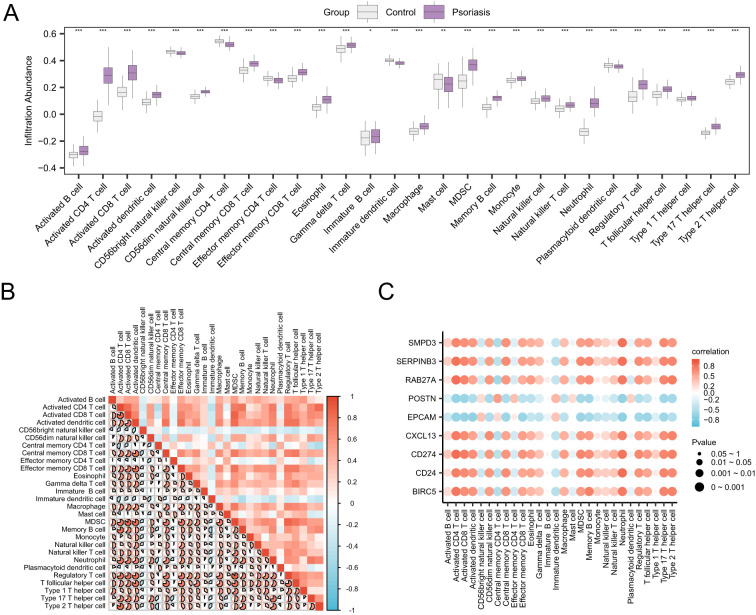
Immune infiltration analysis by ssGSEA algorithm. **(A)** Group comparison of immune cells in the Control and Psoriasis cohorts of the Combined GEO Datasets. **(B)** Correlation heatmap of immune cell infiltration abundance in the Combined GEO Datasets. **(C)** Bubble plot of the correlation between Hub Genes and immune cell infiltration abundance in the integrated GEO Datasets (Combined Datasets). ssGSEA, single-sample Gene-Set Enrichment Analysis; Psoriasis, Psoriasis. * represents a p value < 0.05, which is statistically significant; ** represents p value < 0.01, highly statistically significant; *** represents p value < 0.001 and highly statistically significant. The absolute value of correlation coefficient (r value) below 0.3 was weak or no correlation, 0.3 to 0.5 was weak correlation, 0.5 to 0.8 was moderate correlation, and above 0.8 was strong correlation. Gray was the Control group, and purple was the Psoriasis group. Red is positive correlation, blue is negative correlation. The depth of the color represents the strength of the correlation.

The expression matrices from the Combined Datasets were used to calculate the abundance of immune cell infiltration for 28 immune cells using the ssGSEA algorithm. First, we displayed group comparison plots showing differences in immune cell infiltration abundance among various groups. The group comparison diagram (**Figure 9A**) indicated that all 28 immune cells were statistically significant (*p*< 0.05), including: activated B cell, activated CD4^+^ T cell, activated CD8^+^ T cell, activated dendritic cell, CD56 bright natural killer cell, CD56 dim natural killer cell, central memory CD4^+^ T cell, central memory CD8^+^ T cell, effector memory CD4^+^ T cell, effector memory CD8^+^ T cell, eosinophil, gamma delta T cell, immature B cell, immature dendritic cell, macrophage, mast cell, MDSC, memory B cell, monocyte, natural killer cell, natural killer T cell, neutrophil, plasmacytoid dendritic cell, regulatory T cell (Treg), T follicular helper cell (Tfh), type 1 T helper cell, type 17 T helper cell, and type 2 T helper cell. Next, a correlation heatmap illustrated the results of immune cell infiltration abundance from the immune infiltration analysis in the Combined GEO Datasets (**Figure 9B**). The results indicated that most immune cells exhibited strong positive correlations, with regulatory T cells (Treg) and MDSC showing the most significant correlation (*r* = 0.877, *p*< 0.05). Finally, the correlation between Hub Genes and the abundance of immune cell infiltration was shown by a correlation bubble plot (**Figure 9C**).The correlation bubble plot results indicated that most immune cells displayed strong correlations, with CD274 and activated CD4^+^ T cells showing the most significant positive correlation (*r* = 0.829, *p*< 0.05).

## 4 Discussion

Psoriasis is a chronic skin condition that significantly impacts people worldwide, caused by a complex interaction of genetic, environmental, and immune factors. Key genetic loci such as *HLA-C, TNIP1, IL12B*, and *IL23R (* 29) are linked to psoriasis. However, these genes do not completely account for the disease. This suggests that additional genetic and environmental interactions are at play. Current treatments, such as corticosteroids and biologics, provide relief from psoriasis symptoms, but they have limitations (30). There are still research gaps in understanding genetic mechanisms, exploring gene-environment interactions, and developing effective animal models. Improved research efforts will facilitate the development of more effective treatments and enhance patients’ quality of life. Recent studies suggest that exosomes play a significant role in the pathogenesis of psoriasis. Exosomes are small vesicles released by cells and can carry various bioactive molecules such as cytokines, proteins, and nucleic acids. They regulate cellular functions by interacting with other cells and delivering these bioactive substances (31–33).

Exosomes contain bioactive molecules that can influence skin inflammation, immune responses, and cell proliferation in psoriasis. Exosomes can alleviate inflammatory and autoimmune responses by directly inhibiting the immune system. Research indicates that injecting exosomes into skin lesions of psoriasis patients may reduce inflammation and promote skin repair (34). Analysis of DEGs related to exosomes identified 16 DEGs associated with psoriasis. The identified DEGs include: *CD274 (Programmed Death-Ligand 1, PD-L1), SERPINB3, CD24, RAB27A, RAB27B, BIRC5 (Survivin), CXCL13, SDC4 (Syndecan-4), LGALS3BP (Galectin-3-binding protein), POSTN (Periostin), SMPD3 (Sphingomyelin phosphodiesterase 3), IVL (Involucrin), PLCB4 (Phospholipase C beta 4), EPCAM (Epithelial cell adhesion molecule), FCGBP (Fibrinogen C domain-containing protein 6, Keratinocyte differentiation-associated protein 1), and MYOC (Myocilin).* The expression of these genes is closely associated with the pathogenesis of psoriasis. *CD274* is an immune regulatory protein that is widely believed to play an important role in immune tolerance and immune evasion. In psoriasis, the expression of *CD274* may regulate the activation of immune cells and inflammatory responses, thus influencing the development of psoriasis (35, 36).

Some studies indicate that *CD274 (PD-L1), CXCL13, BIRC5*, and *SMPD3* are important in the pathogenesis of psoriasis. *CD274* and *CXCL13* regulate immune cell activity and inflammatory responses. *BIRC5* regulates cell apoptosis and survival, while *SMPD3* is involved in intracellular transport and cell signaling (37–40). Abnormal expression of these genes is associated with inflammation, cell proliferation, and immune system dysregulation in psoriasis. *CD274 (PD-L1), CXCL13, BIRC5*, and *SMPD3* play the following roles in the *IL-17a* and *IL-23* pathways: *CD274 (PD-L1)*: The *IL-17a* and *IL-23* pathways are closely related to the pathogenesis of autoimmune diseases like psoriasis. *CD274* is the ligand of *PD-1*, and its expression is regulated by *IL-17a* and *IL-23*. *IL-23* induces the differentiation of T cells into *Th17* cells, which produce *IL-17a. IL-17a* regulates the expression level of *PD-L1* on immune cells. When *PD-L1* binds to *PD-1*, it inhibits T cell immune activity. Therefore, *CD274* (*PD-L1*) may be involved in regulating the activity of the *IL-17a* and *IL-23* pathways in psoriasis, affecting autoimmune reactions and the occurrence of inflammation. *CXCL13* is a chemokine that plays a crucial role in inflammation. *IL-17a* and *IL-23* may affect the migration of inflammatory cells and the occurrence of inflammation by regulating the expression of *CXCL13*. Thus, *CXCL13* may mediate the aggregation of inflammatory cells, contributing to the exacerbation of inflammation in the *IL-17a* and *IL-23* pathways. The *IL-17a* and *IL-23* pathways are associated with cell proliferation and survival. *BIRC5* is a gene that encodes a protein called survivin, and its abnormal expression in psoriasis may be related to the regulation of cell apoptosis and survival in the *IL-17a* and *IL-23* pathways. The signaling of *IL-17a* and *IL-23* may lead to the overexpression of *BIRC5*, inhibiting apoptosis and resulting in abnormal proliferation of keratinocytes and keratinization. *SMPD3* may be involved in the signaling and intracellular substance transport process of the *IL-17a* and *IL-23* pathways. *SMPD3* encodes an acid sphingomyelinase involved in intracellular substance transport and cell signaling processes. The *IL-17a* and *IL-23* pathways may regulate the expression of *SMPD3* in immune cell activation and inflammatory response, affecting cell signaling and the production of inflammatory mediators. *IL-17a* and *IL-23* are two cytokines closely associated with the pathogenesis of autoimmune diseases, including psoriasis. They play important roles in processes such as inflammatory response, regulation of immune cells, and abnormal proliferation of keratinocytes. *IL-17a* promotes inflammation and abnormal proliferation of keratinocytes. This leads to characteristic symptoms such as redness, scaling, and itching in affected areas. On the other hand, *IL-23* affects immune cell differentiation and promotes the production of pro-inflammatory mediators.*CD274 (PD-L1)* is an immune regulatory protein believed to play a crucial role in immune tolerance and immune evasion. Our study found a strong link between *CD274* and the development of psoriasis. This indicates its role in regulating immune cell activation and inflammatory responses. This is consistent with our findings and opens up opportunities to further explore *CXCL13’s* role in psoriasis pathophysiology. *CXCL13* is a chemokine involved in regulating inflammatory responses and immune cell migration. Our study found that *CXCL13* is related to the pathogenesis of psoriasis, confirming its significance in modulating inflammation and cell migration. *BIRC5 (Survivin)* is an anti-apoptotic protein thought to play a critical role in cell survival and apoptosis (41–44). Our study revealed the association of *BIRC5* with the pathogenesis of psoriasis, suggesting its potential involvement in regulating cell apoptosis and survival in psoriasis. This is consistent with our study results and provides clues for further research into the functions and mechanisms of *BIRC5* in the development of psoriasis. *SMPD3* is a lysosomal enzyme involved in intracellular signal transduction and regulating cellular lipid metabolism. In our study, we found that *SMPD3* is closely related to the pathogenesis of psoriasis, indicating its importance in cellular signal transduction and lipid metabolism regulation. This is consistent with recent research findings and points to a new direction for investigating the functions and mechanisms of *SMPD3*. It is noteworthy that this study not only focuses on *SMPD3* but also establishes, for the first time, a connection between *CD274, CXCL13*, *BIRC5*, and the development of psoriasis, suggesting that they may regulate processes such as cell activation, inflammation, and apoptosis in the *IL-17a* and *IL-23* signaling pathways. For example, recent studies have revealed changes in the expression of key apoptosis-related genes in the pathogenesis of psoriasis. Specifically, *BIRC5 (Survivin)*, an anti-apoptotic protein, was found to be significantly upregulated in psoriatic lesions, suggesting its important role in regulating cell apoptosis and proliferation. Additionally, studies have shown that other genes such as *FAS, BAX*, and *BCL-2* also exhibit different expression patterns in the skin of psoriasis patients, and these genes are directly involved in the regulation of apoptosis. Modulating the expression of these apoptosis-related genes may provide new targets for the treatment of psoriasis. At the same time, research on immune infiltration further reveals the complexity of psoriasis. Recent single-cell RNA sequencing analyses show that various immune cells, including T cells, B cells, and macrophages, infiltrate psoriatic lesions. This infiltration is closely linked to inflammation levels. Specific chemokines (such as *CXCL13*) play a crucial role in the recruitment of these immune cells, further exacerbating the inflammatory state of the lesions. These research findings highlight the interplay between immune cell infiltration, insulin resistance, apoptosis, and survival signals, forming an important network in the pathophysiology of psoriasis. In summary, our research establishes, for the first time, the relationships between *SMPD3, CD274, CXCL13*, and *BIRC5.* It also integrates relevant gene expression studies, highlighting the complexity of apoptosis and immune infiltration in psoriasis pathogenesis. These findings provide a solid foundation for further understanding the pathophysiology of psoriasis and open new avenues for developing novel therapeutic targets (45–48).

We must acknowledge the limitations of our study. First, our research focused solely on differentially expressed genes in exosomes related to psoriasis, neglecting gene changes from other cellular sources and their roles in psoriasis pathogenesis. Second, further investigation and validation are needed to clarify the functions and regulatory mechanisms of these genes in psoriasis development. Moreover, our study’s small sample size may lead to variability, highlighting the need for validation in larger studies. Acknowledging these limitations will promote future research to enhance the reliability and generalizability of our findings.

## 5 Conclusions

Exosomes play a significant role in the pathogenesis of psoriasis. They regulate cellular functions by interacting with other cells and delivering bioactive molecules. The bioactive molecules in exosomes contribute to the regulation of skin inflammation, immune response, and cell proliferation, all of which are pathological processes in psoriasis. They can directly inhibit the immune response, which helps reduce inflammation and autoimmune reactions. Further research is needed to uncover the specific details of their actions and interactions.

## Data Availability

The datasets presented in this study can be found in online repositories. The names of the repository/repositories and accession number(s) can be found in the article/**Supplementary Material**.

## References

[1] Griffiths CEM Armstrong AW Gudjonsson JE Barker JNWN. Psoriasis. Lancet. (2021) 397:1301–15. doi: 10.1016/S0140-6736(20)32549-6 33812489

[2] Lowes MA Suárez-Fariñas M Krueger JG. Immunology of psoriasis. Annu Rev Immunol. (2014) 32:227–55. doi: 10.1146/annurev-immunol-032713-120225 PMC4229247 24655295

[3] Yu Z Yu Q Xu H Dai X Yu Y Cui L. IL-17A promotes psoriasis-associated keratinocyte proliferation through ACT1-dependent activation of YAP-AREG axis. J Invest Dermatol. (2022) 142:2343–52. doi: 10.1016/j.jid.2022.02.016 35304250

[4] Parisi R Iskandar IYK Kontopantelis E Augustin M Griffiths CEM Ashcroft DM. National, regional, and worldwide epidemiology of psoriasis: systematic analysis and modelling study. Bmj. (2020) 369:m1590. doi: 10.1136/bmj.m1590 32467098 PMC7254147

[5] Menter AM Korman NJ Elmets CA Feldman SR Gelfand JM Gordon KB. Guidelines of care for the management of psoriasis and psoriatic arthritis: section 6. Guidelines of care for the treatment of psoriasis and psoriatic arthritis: case-based presentations and evidence-based conclusions. J Am Acad Dermatol. (2011) 65:137–74. doi: 10.1016/j.jaad.2010.11.055 21306785

[6] Nyholm N Schnack H Danø A Skowron F. Cost per responder of biologic drugs used in the treatment of moderate-to-severe plaque psoriasis in France and Germany. Curr Med Res Opin. (2023) 39:833–42. doi: 10.1080/03007995.2023.2214046 37203343

[7] Khan S Bennit HF Wall NR. The emerging role of exosomes in survivin secretion. Histol Histopathol. (2015) 30:43–50. doi: 10.14670/HH-30.43 25020159 PMC4489405

[8] Gurung S Perocheau D Touramanidou L Baruteau J. The exosome journey: from biogenesis to uptake and intracellular signalling. Cell Commun Signal. (2021) 19:47. doi: 10.1186/s12964-021-00730-1 33892745 PMC8063428

[9] Barile L Vassalli G. Exosomes: Therapy delivery tools and biomarkers of diseases. Pharmacol Ther. (2017) 174:63–78. doi: 10.1016/j.pharmthera.2017.02.020 28202367

[10] Davis S Meltzer PS. GEOquery: a bridge between the gene expression omnibus (GEO) and bioConductor. Bioinformatics. (2007) 23:1846–7. doi: 10.1093/bioinformatics/btm254 17496320

[11] Suárez-Fariñas M Li K Fuentes-Duculan J Hayden K Brodmerkel C Krueger JG. Expanding the psoriasis disease profile: interrogation of the skin and serum of patients with moderate-to-severe psoriasis. J Invest Dermatol. (2012) 132:2552–64. doi: 10.1038/jid.2012.184 PMC3472561 22763790

[12] Swindell WR Johnston A Carbajal S Han G Wohn C Lu J. Genome-wide expression profiling of five mouse models identifies similarities and differences with human psoriasis. PloS One. (2011) 6:e18266. doi: 10.1371/journal.pone.0018266 21483750 PMC3070727

[13] Stelzer G Rosen N Plaschkes I Zimmerman S Twik M Fishilevich S. The geneCards suite: from gene data mining to disease genome sequence analyses. Curr Protoc Bioinf. (2016) 54:1.30.1–1.30.33. doi: 10.1002/0471250953.2016.54.issue-1 27322403

[14] Lin Y Huang K Cai Z Chen Y Feng L Gao Y. A novel exosome-relevant molecular classification uncovers distinct immune escape mechanisms and genomic alterations in gastric cancer. Front Pharmacol. (2022) 13:884090. doi: 10.3389/fphar.2022.884090 35721114 PMC9204030

[15] Leek JT Johnson WE Parker HS Jaffe AE Storey JD. The sva package for removing batch effects and other unwanted variation in high-throughput experiments. Bioinformatics. (2012) 28:882–3. doi: 10.1093/bioinformatics/bts034 PMC3307112 22257669

[16] Ritchie ME Phipson B Wu D Hu Y Law CW Shi W. limma powers differential expression analyses for RNA-sequencing and microarray studies. Nucleic Acids Res. (2015) 43:e47. doi: 10.1093/nar/gkv007 25605792 PMC4402510

[17] Ben Salem K Ben Abdelaziz A. Principal component analysis (PCA). Tunis Med. (2021) 99(4):383–9. PMC8734479 35244921

[18] Zhang H Meltzer P Davis S. RCircos: an R package for Circos 2D track plots. BMC Bioinf. (2013) 14:244. doi: 10.1186/1471-2105-14-244 PMC3765848 23937229

[19] Mi H Muruganujan A Ebert D Huang X Thomas PD. PANTHER version 14: more genomes, a new PANTHER GO-slim and improvements in enrichment analysis tools. Nucleic Acids Res. (2019) 47:D419–d426. doi: 10.1093/nar/gky1038 30407594 PMC6323939

[20] Ogata H Goto S Sato K Fujibuchi W Bono H Kanehisa M. KEGG: kyoto encyclopedia of genes and genomes. Nucleic Acids Res. (2000) 28:27–30. doi: 10.1093/nar/28.1.27 9847135 PMC148090

[21] Yu G Wang LG Han Y He QY. clusterProfiler: an R package for comparing biological themes among gene clusters. Omics. (2012) 16:284–7. doi: 10.1089/omi.2011.0118 PMC3339379 22455463

[22] Subramanian A Tamayo P Mootha VK Mukherjee S Ebert BL Gillette MA. Gene set enrichment analysis: a knowledge-based approach for interpreting genome-wide expression profiles. Proc Natl Acad Sci U S A. (2005) 102:15545–50. doi: 10.1073/pnas.0506580102 PMC1239896 16199517

[23] Szklarczyk D Gable AL Lyon D Junge A Wyder S Huerta-Cepas J. STRING v11: protein-protein association networks with increased coverage, supporting functional discovery in genome-wide experimental datasets. Nucleic Acids Res. (2019) 47:D607–d613. doi: 10.1093/nar/gky1131 30476243 PMC6323986

[24] Shannon P Markiel A Ozier O Baliga NS Wang JT Ramage D. Cytoscape: a software environment for integrated models of biomolecular interaction networks. Genome Res. (2003) 13:2498–504. doi: 10.1101/gr.1239303 PMC403769 14597658

[25] Chin CH Chen SH Wu HH Ho CW Ko MT Lin CY. cytoHubba: identifying hub objects and sub-networks from complex interactome. BMC Syst Biol. (2014) 8 Suppl 4:S11. doi: 10.1186/1752-0509-8-S4-S11 25521941 PMC4290687

[26] Zhou KR Liu S Sun WJ Zheng LL Zhou H Yang JH. ChIPBase v2.0: decoding transcriptional regulatory networks of non-coding RNAs and protein-coding genes from ChIP-seq data. Nucleic Acids Res. (2017) 45:D43–d50. doi: 10.1093/nar/gkw965 27924033 PMC5210649

[27] Li JH Liu S Zhou H Qu LH Yang JH. starBase v2.0: decoding miRNA-ceRNA, miRNA-ncRNA and protein-RNA interaction networks from large-scale CLIP-Seq data. Nucleic Acids Res. (2014) 42:D92–7. doi: 10.1093/nar/gkt1248 PMC3964941 24297251

[28] Xiao B Liu L Li A Xiang C Wang P Li H. Identification and verification of immune-related gene prognostic signature based on ssGSEA for osteosarcoma. Front Oncol. (2020) 10:607622. doi: 10.3389/fonc.2020.607622 33384961 PMC7771722

[29] Tsoi LC Spain SL Knight J Ellinghaus E Stuart PE Capon F. Identification of 15 new psoriasis susceptibility loci highlights the role of innate immunity. Nat Genet. (2012) 44:1341–8. doi: 10.1038/ng.2467 PMC3510312 23143594

[30] Palmer V Cornier MA Waring A Valdebran M. Evaluation and treatment of metabolic syndrome and cardiovascular disease in adult patients with psoriasis. Int J Dermatol. (2023) 62:1437–46. doi: 10.1111/ijd.v62.12 37845786

[31] Mirzaei R Zamani F Hajibaba M Rasouli-Saravani A Noroozbeygi M Gorgani M. The pathogenic, therapeutic and diagnostic role of exosomal microRNA in the autoimmune diseases. J Neuroimmunol. (2021) 358:577640. doi: 10.1016/j.jneuroim.2021.577640 34224949

[32] Jonoush ZA Mahdavi R Farahani M Zeinali F Shayan E Amari A. The implications of exosomes in psoriasis: disease: emerging as new diagnostic markers and therapeutic targets. Mol Biol Rep. (2024) 51:465. doi: 10.1007/s11033-024-09449-x 38551769

[33] Tang B Bi Y Zheng X Yang YJ Huang XB Yang KX. The role of extracellular vesicles in the development and treatment of psoriasis: narrative review. Pharmaceutics. (2024) 16(12):1586. doi: 10.3390/pharmaceutics16121586 39771564 PMC11677080

[34] Yu HH Feng H Zeng H Wu YP Zhang Q Yu J. Exosomes: The emerging mechanisms and potential clinical applications in dermatology. Int J Biol Sci. (2024) 20:1778–95. doi: 10.7150/ijbs.92897 PMC10929203 38481799

[35] Kim JY Park M Kim YH Ryu KH Lee KH Cho KA. Tonsil-derived mesenchymal stem cells (T-MSCs) prevent Th17-mediated autoimmune response via regulation of the programmed death-1/programmed death ligand-1 (PD-1/PD-L1) pathway. J Tissue Eng Regener Med. (2018) 12:e1022–33. doi: 10.1002/term.v12.2 28107610

[36] Tanaka R Ichimura Y Kubota N Saito A Nakamura Y Ishitsuka Y. Differential involvement of programmed cell death ligands in skin immune responses. J Invest Dermatol. (2022) 142:145–154.e8. doi: 10.1016/j.jid.2021.06.026 34310947

[37] Zdanowska N Kasprowicz-Furmańczyk M Placek W Owczarczyk-Saczonek A. The role of chemokines in psoriasis-an overview. Medicina (Kaunas). (2021) 57(8):754. doi: 10.3390/medicina57080754 34440960 PMC8400543

[38] Sieminska I Pieniawska M Grzywa TM. The immunology of psoriasis-current concepts in pathogenesis. Clin Rev Allergy Immunol. (2024) 66:164–91. doi: 10.1007/s12016-024-08991-7 PMC11193704 38642273

[39] Burger B Sagiorato RN Cavenaghi I Rodrigues HG. Abnormalities of sphingolipids metabolic pathways in the pathogenesis of psoriasis. Metabolites. (2023) 13(2):291. doi: 10.3390/metabo13020291 36837912 PMC9968075

[40] Zhuang H Wang XM Guo M Meng QQ Liu N Wei M. Identification of chemokines-related miRNAs as potential biomarkers in psoriasis based on integrated bioinformatics analysis. Comb Chem High Throughput Screen. (2023) 26:1400–13. doi: 10.2174/1386207325666220819194249 PMC10234082 35993468

[41] Hui L Li Y Huang MK Jiang YM Liu T. CXCL13: a common target for immune-mediated inflammatory diseases. Clin Exp Med. (2024) 24:244. doi: 10.1007/s10238-024-01508-8 39443356 PMC11499446

[42] Zou A Chen Y Liu TS Yang T Zhou B. Identification and verification of three autophagy-related genes as potential biomarkers for the diagnosis of psoriasis. Sci Rep. (2023) 13:22918. doi: 10.1038/s41598-023-49764-0 38129460 PMC10739819

[43] Borodzicz S Rudnicka L Mirowska-Guzel D Cudnoch-Jedrzejewska A. The role of epidermal sphingolipids in dermatologic diseases. Lipids Health Dis. (2016) 15:13. doi: 10.1186/s12944-016-0178-7 26786937 PMC4717587

[44] Boutet MA Nerviani A Gallo Afflitto G Pitzalis C. Role of the IL-23/IL-17 axis in psoriasis and psoriatic arthritis: the clinical importance of its divergence in skin and joints. Int J Mol Sci. (2018) 19(2):530. doi: 10.3390/ijms19020530 29425183 PMC5855752

[45] Zou A Kong Q Sang H. Identification of key apoptosis-related genes and immune infiltration in the pathogenesis of psoriasis. Hereditas. (2022) 159:26. doi: 10.1186/s41065-022-00233-0 35729678 PMC9213172

[46] Huang YH See LC Chang YC Chung WH Chang LC Yang SF. Impact of ABCG2 gene polymorphism on the predisposition to psoriasis. Genes (Basel). (2021) 12(10):1601. doi: 10.3390/genes12101601 34680995 PMC8535938

[47] Puig L Julià A Marsal S. The pathogenesis and genetics of psoriasis. Actas Dermosifiliogr. (2014) 105:535–45. doi: 10.1016/j.ad.2012.11.006 23369832

[48] Liu TM Li S Ying S Tang S Ding Y Li Y. The IL-23/IL-17 pathway in inflammatory skin diseases: from bench to bedside. Front Immunol. (2020) 11:594735. doi: 10.3389/fimmu.2020.594735 33281823 PMC7705238

